# Bovine Colostrum Before or After Formula Feeding Improves Systemic Immune Protection and Gut Function in Newborn Preterm Pigs

**DOI:** 10.3389/fimmu.2019.03062

**Published:** 2020-01-30

**Authors:** Yanqi Li, Xiaoyu Pan, Duc Ninh Nguyen, Shuqiang Ren, Arshnee Moodley, Per Torp Sangild

**Affiliations:** ^1^Comparative Pediatrics and Nutrition, Department of Veterinary and Animal Sciences, University of Copenhagen, Copenhagen, Denmark; ^2^Veterinary Clinical Microbiology, Department of Veterinary and Animal Sciences, University of Copenhagen, Copenhagen, Denmark; ^3^Department of Pediatrics and Adolescent Medicine, Rigshospitalet, Copenhagen, Denmark; ^4^Department of Paediatrics, Odense University Hospital, Odense, Denmark

**Keywords:** bovine colostrum, formula feeding, preterm infants, systemic immunity, gut health, necrotizing enterocolitis

## Abstract

**Objectives:** Maternal milk is often absent or in limited supply just after preterm birth. Many preterm infants are therefore fed infant formula as their first enteral feed despite an increased risk of feeding intolerance, necrotizing enterocolitis (NEC), and infection. Using preterm pigs as a model for preterm infants, we hypothesized that bovine colostrum given before or after formula feeding would alleviate formula-induced detrimental effects during the first days after preterm birth.

**Methods:** A total of 74 preterm pigs received gradually increasing volumes of formula (F) or bovine colostrum (C) until day 5, when they were euthanized or transitioned to either C or F for another 4 days, resulting in six groups: C or F until day 5 (C5, F5, *n* = 11 each), C or F until day 9 (CC, FF *n* = 12–13 each), C followed by F (CF, *n* = 14), and F followed by C (FC, *n* = 13).

**Results:** Systemically, colostrum feeding stimulated circulating neutrophil recruitment on day 5 (C5 vs. F5, *P* < 0.05). Relative to initial formula feeding, initial colostrum feeding promoted the development of systemic immune protection as indicated by a decreased T-helper cell population and an increased regulatory T-cell population (CC + CF vs. FC + FF, *P* < 0.01). In the gut, colostrum feeding improved intestinal parameters such as villus heights, enzymes, hexose absorption, colonic goblet cell density, and decreased the incidence of severe NEC (27 vs. 64%), diarrhea (16 vs. 49%), and gut permeability on day 5, coupled with lowered expression of *LBP, MYD88, IL8, HIF1A*, and *CASP3* (C5 vs. F5, all *P* < 0.05). On day 9, the incidence of severe NEC was similarly low across groups (15–21%), but diarrhea resistance and intestinal parameters were further improved by colostrum feeding, relative to exclusive formula feeding (CC, CF, or FC vs. FF, respectively, all *P* < 0.05). The expression of *MYD88* and *CASP3* remained downregulated by exclusive colostrum feeding (CC vs. FF, *P* < 0.01) and colostrum before or after formula feeding down regulated *HIF1A* and *CASP3* expression marginally.

**Conclusion:** Colostrum feeding ameliorated detrimental effects of formula feeding on systemic immunity and gut health in preterm newborns, especially when given immediately after birth.

## Introduction

Optimal nutrition supply is an essential aspect of clinical care for very preterm infants (gestational age < 32 weeks). Clinical nutrition guidelines recommend initiation of enteral feeding within a few hours, and the goal is to minimize time to full enteral feeding (TFF) without inducing feeding intolerance and other intestinal complications, such as necrotizing enterocolitis (NEC) (1–4). This goal is better achieved when maternal milk (MM) is available. Unfortunately, it is often seen that MM is in short supply immediately after very preterm birth, and preterm infant formula (indicated as formula below), which is designed to mimic MM, is therefore used as the first milk, particularly in hospitals with limited resources (e.g., space for mothers, personnel and freezers to receive expressed MM, and donor milk banks). However, formula remains associated with longer TFF, increased risk of feeding intolerance and NEC (5), and impaired systemic immunity (e.g., more sepsis) (6, 7). This may be due to the fact that formula lacks many bioactive components that are present in MM and beneficial to immune defense (e.g., alpha-lactalbumin, lactoferrin, osteopontin) (8). Natural MM also contains various sugars (e.g., lactose, glucose, and oligosaccharides), while formula contains high levels of plant-derived carbohydrates (e.g., maltodextrin), which may impair antibacterial responses (9). In addition, milk fat is replaced by vegetable oil during formula processing, and therefore, formula also lacks milk fat globule membrane, which consists of numerous lipids and proteins that have functions on immune-related pathways (10, 11).

Early feeding of formula, even with small volumes for a short period, has showed detrimental effects in the gut (12, 13). Hence, it is crucial to find an alternative to formula as the first milk especially in the resource-limited hospitals. Bovine colostrum (indicated as colostrum below), the first milk from cows postpartum, contains high levels of trophic, antimicrobial, and immune-modulatory factors, such as insulin-like growth factor-I (IGF-I), immunoglobulins, lysozymes, transforming growth factor-β (TGF-β), and lactoferrin, similar to the bioactive components found in MM (14). It has been repeatedly shown that, in preterm pigs, a model for preterm infants, colostrum improves digestive and absorptive functions, dampens inflammation, protects against NEC, and improves systemic immunity, relative to formula (12, 13, 15–19). Therefore, we investigated whether initial colostrum feeding protects against later suboptimal formula feeding and whether colostrum has any therapeutic effects on an impaired intestine caused by initial formula feeding. We hypothesized that colostrum provided either before or after formula feeding just after preterm birth protected against formula-induced detrimental effects on systemic immunity and gut health. Using preterm pigs as a model for preterm infants, we fed different combinations of colostrum and isocaloric formula for 9 days and measured clinical variables, blood biochemistry and cell counts, gut functions and NEC incidence, systemic immunity, and gut immune gene expressions.

## Methods

### Pigs, Experimental Design, and Nutrition

All animal procedures were approved by the Danish National Committee on Animal Experimentation. Seventy-four preterm pigs were delivered from four sows by cesarean section at 106 days gestation (Large White Danish × Landrace × Duroc, Askelygaard Farm; term = 117 ± 2 days; male/female, 36/38). Surgical procedures were performed with an orogastric feeding tube and an umbilical catheter for parental nutrition and sow's plasma (for passive immunization), as described previously (20). The pigs were stratified according to birth weight and gender and allocated randomly into two groups: one group receiving colostrum and the other group receiving formula, both for 4 days until day 5 of the experiment. On day 4, pigs in each group were further stratified according to body weight and gender and allocated randomly into three groups: euthanasia on day 5, continuation with the same feeding for another 4 days, and shift to the other diet for an additional 4 days. For pigs euthanized on day 5, there were two groups: colostrum feeding until day 5 (C5, *n* = 11) and formula feeding until day 5 (F5, *n* = 11). For pigs euthanized on day 9, there were four feeding groups: colostrum feeding until day 9 (CC, *n* = 12), colostrum feeding for 4 days followed by formula until day 9 (CF, *n* = 14), formula feeding for 4 days followed by colostrum until day 9 (FC, *n* = 13), and formula feeding until day 9 (FF, *n* = 13). For repeated variables measured before euthanasia on day 5, pigs fed colostrum and formula were termed as C and F, respectively (*n* = 37 each). A sample size of 10–15 piglets per group is often used in this model to detect a ~50% reduction in NEC incidence (α = 0.05, β = 80%), and this reduction is expected when comparing bovine colostrum and infant formula feeding according to our previous studies (21).

**Figure 1:**
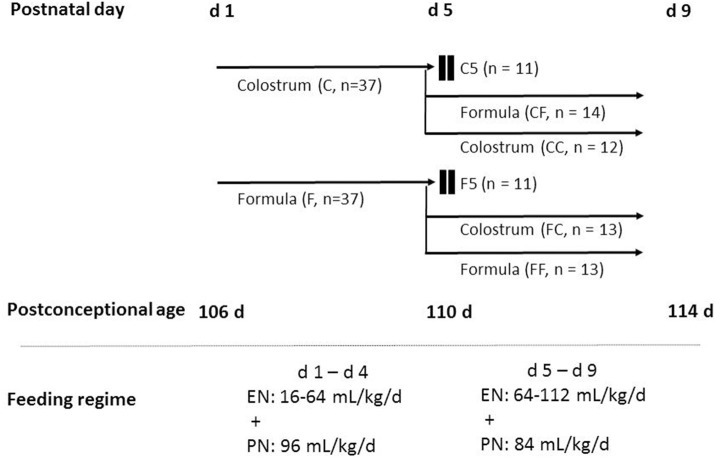
Schematic overview of the animal experimental design. Seventy-four preterm pigs from four sows were delivered at 106 days gestation. The pigs were stratified according to birth weight and gender and allocated randomly into two groups: one group receiving colostrum (C, *n* = 37) and the other group receiving formula (F, *n* = 37) for 4 days until day 5 of the experiment. On day 4, pigs in each group were further stratified into three groups to be euthanized on day 5, fed the same feeding for another 4 days, and fed the other diet for another 4 days resulting in six groups: colostrum feeding until day 5 (C5, *n* = 11), formula feeding until day 5 (F5, *n* = 11), colostrum feeding for 4 days followed by formula until day 9 (CF, *n* = 14), colostrum feeding until day 9 (CC, *n* = 12), formula feeding for 4 days followed by colostrum until day 9 (FC, *n* = 13), and formula feeding until day 9 (FF, *n* = 13). Pigs received gradually increasing volumes of enteral nutrition 16–64 ml kg^−1^ day^−1^ on days 1–4 and 64–112 ml kg^−1^ day^−1^ on days 5–8. Parenteral nutrition was given at decreasing rates of 96–84 ml kg^−1^ day^−1^. C, colostrum; EN, enteral nutrition; F, formula; PN, parenteral nutrition.

Pigs received gradually increasing volumes of enteral nutrition from 16 ml kg^−1^ day^−1^ at birth to 64 ml kg^−1^ day^−1^ on day 4 (increasing by 16 ml kg^−1^ day^−1^) and volumes were kept at this level on day 5 and increased gradually again to 112 ml kg^−1^ day^−1^ on day 8 (increasing by 16 ml kg^−1^ day^−1^). The colostrum diet was freshly prepared each day by reconstitution of 170 g colostrum powder (ColoDan, Biofiber Damino, Gesten, Denmark) into 1 L water and stored at 4°C. The formula diet was prepared by blending the following commercially available ingredients, providing protein (whey, DI-9224 whey protein isolate; casein, Miprodan 40; both from Arla Foods Ingredients, Århus, Denmark), carbohydrate (Fantomalt, from Nutricia, Allerøde, Denmark), lipids (Liquigen, Calogen; Nutricia), and vitamins and minerals (SHS Seravit; Nutricia). The amounts of each ingredient were adjusted to ensure the same macronutrient composition and energy levels for the colostrum and formula diets (Table 1). Before each feeding, diets were warmed in a water bath not exceeding 40°C. Parental nutrition was given to maintain sufficient amount of fluid and nutrients. The rate was 96 ml kg^−1^ day^−1^ for the first 4 days and 84 ml kg^−1^ day^−1^ for the remaining days. If the catheters dislocated before euthanasia, enteral nutrition was accordingly increased. A commercially available parenteral nutrition product (Kabiven, Fresenius Kabi, Bad Homburg, Germany) was used after adjustments, as earlier described (22). The experimental design is illustrated in Figure 1.

**Table 1:** Nutrient composition of experimental diets.

**Constituent L**	**Colostrum**	**Formula**
SHS Seravit (g)		12
SHS Liquigen (100 ml)		43
Nutricia Calogen (100 ml)		30
Nutricia Fantomalt (g)		18
Whey protein isolate DI-9224 (g)		70
Casein Miprodan 40 (g)		35
Colondan colostrum powder (g)	170	
Energy (kJ/L)	3,405	3,398
Protein (g/L)	92.2	92.4
Whey (g/L)	62	62
Casein (g/L)	30	30
Fat (g/L)	37.4	37.1
Carbohydrate (g/L)	26.7	26.5
Maltodextrin		24
Lactose (g/L)	26.7	
Others (g/L)		2
Minerals (g/L)		
Calcium	1.70	0.41
Phosphorus	1.53	0.35
Iron (mg/L)	0	8
Sodium	0.51	0.79
Magnesium	0.34	0.04
Potassium	1.02	0.91

### Clinical Evaluation and Sample Collection

Pigs were continually monitored and were euthanized with an intracardiac injection of pentobarbitone sodium (60 mg/kg) upon clinical indications (e.g., severe pain) or at the end of the study for sample collection. Clinical conditions, fecal characters (e.g., firm, pasty, diarrhea, bloody diarrhea), and degree of dehydration were recorded twice daily according to predefined scoring systems (18). Body weights were measured once a day, and daily weight gain was calculated. Time to motor skill acquisition was monitored every 3 h until the pigs were able to open at least one eye, to stand without support, or to walk without support. The physical activity of each pig was recorded by infrared video surveillance cameras placed over each incubator to analyze the proportion of active time using a motion-detection software (PigLWin, Ellegaard Systems, Faaborg, Denmark).

At euthanasia, organ weights of lungs, heart, liver, spleen, kidneys, adrenals, stomach, small intestine (SI), and colon were measured. The entire SI was evenly divided into three regions, proximal (Prox), middle (Mid), and distal (Dist), and the length was measured. Macroscopic lesion scores (1–6) were given to stomach, Prox, Mid, Dist, and colon, respectively, depending on the extent and severity of pathological changes such as hyperemia, edema, hemorrhage, pneumatosis, and necrosis as previously described (18). Pigs with a score of ≥3 in any of the four intestinal regions were diagnosed as “NEC,” while pigs with a score of ≥4 were identified as “severe NEC.” Intestinal whole-wall and mucosal tissue samples were collected from each region and immediately snap frozen in liquid nitrogen and stored at −80°C or fixed in paraformaldehyde solution for further analyses. The intact left thigh of each pig was dislocated, without breaking any bones for subsequent bacterial analysis of the bone marrow. Bacteria were cultured from aseptically collected bone marrow samples from the left femur and cerebrospinal fluid (CSF) on 5% blood agar and colonies were enumerated, isolated, and identified using matrix-assisted laser desorption/ionization time-of-flight mass spectrometry as previously described (23).

### Blood Biochemistry, Blood Cell Count, and Systemic Immunity

Blood samples were collected at the time of euthanasia from cardiac blood, and plasma was obtained for the analysis of biochemistry and inflammatory mediators. Blood biochemistry was analyzed by the Advia 1800 Chemistry System (Siemens, Erlangen, Germany). Plasma inflammatory mediators, such as C-reactive protein, interleukin (IL)-1β, and IL-6 at euthanasia were analyzed by commercial porcine DuoSet ELISA kits according to the manufacturer's protocols (R&D Systems Denmark, Abingdon, United Kingdom). On days 5 and 9, arterial blood from the umbilical catheter was collected before euthanasia for the characterization of immune cell subsets and functions, including blood cell count by an automatic cell counter (Advia 2120i Hematology System, Siemens, Germany), T-cell profiling, and *ex vivo* neutrophil phagocytosis function by flow cytometry, as previously described (24, 25). Neutrophil phagocytosis function was measured as the proportion of phagocytic neutrophils using pHrodo Red *Escherichia coli* bioparticles phagocytosis kit for flow cytometry (Thermofisher). Briefly, whole blood was incubated with pHrodo Red-conjugated *E. coli* at ~10:1 particle/phagocyte ratio at 37°C and 5% CO_2_ for 30 min. After removing erythrocytes by the lysis buffer, leukocytes were washed and analyzed on a BD Accuri C6 flow cytometer (BD Biosciences). The proportion of phagocytic neutrophils was calculated as the proportion of pHrodo^+^ neutrophils in the total neutrophil population (%).

For T-cell subset profiling, erythrocytes from whole blood were lysed by BD FACS lysing solution (BD Biosciences, Lyngby, Denmark), and the remaining leukocytes were then washed and permeabilized in fixation/permeabilization buffer (eBioscience, San Diego, CA, USA) for 30 min at 4°C in the dark. Thereafter, Fc receptors were blocked by porcine serum (10 min, 4°C in the dark), and the cells were stained with a mixture of four antibodies: PerCP-Cy5.5 conjugated anti-pig CD3 antibody (IgG2a isotype, BD Biosciences), fluorescein isothiocyanate-conjugated anti-pig CD4 antibody (IgG2b isotype, BioRad, Copenhagen, Denmark), phycoerythrin-conjugated mouse anti-pig CD8 antibody (IgG2a, isotype, Bio-Rad), and allophycocyanin-conjugated antimouse/rat Foxp3 antibody (IgG2a isotype, eBioscience). T-cell subsets were then identified by their markers using a BD Accuri C6 flow cytometer (BD Biosciences), including helper T cells (T_h_, CD3^+^CD4^+^CD8^−^ lymphocytes), cytotoxic T cells (T_c_, CD3^+^CD4^−^CD8^+^ lymphocytes), double-negative T cells (DN, CD3^+^CD4^−^CD8^−^ lymphocytes), and regulatory T cells (T_reg_, CD3^+^CD4^+^Foxp3^+^ lymphocytes).

### Gut Histology, Brush Border Enzyme Activities, and *in vivo* Functions

Villus height and crypt depth of three small intestinal regions (proximal, middle, and distal) were measured on light microscopy images using the Image J processing and analysis software program on HE-stained paraformaldehyde-fixed histology slices (22). Mucin-containing goblet cell density in the distal SI and colon was quantified by Alcian blue and periodic acid–Shiff staining as previously described (26). Activities of six brush border enzymes, lactase, maltase, sucrase, aminopeptidase N (ApN), aminopeptidase A (ApA), dipeptidylpeptidase IV (DPPIV) were analyzed in tissue homogenates (12). Intestinal absorptive function was assessed *in vivo* by measuring the concentration of plasma galactose after an oral bolus of 10% galactose (15 ml/kg) on days 4 and 8. Intestinal permeability was tested *in vivo* by giving an oral bolus (15 ml/kg) containing 5% lactulose and 5% mannitol 3 h before urine collection at euthanasia and measuring its lactulose to mannitol ratio. Detailed methods of these *in vivo* tests have been described earlier (22).

### Intestinal Mucosal Gene Expression

Eleven genes related to gut immunity, hypoxia, apoptosis, angiogenesis, and integrity and a house keeping gene (hypoxanthine-guanine phosphoribosyltransferase, HPRT1) were selected and measured by quantitative PCR. Primers spanning the exon–exon junctions (Table 2) were designed using Primer3 version 0.4.0 (bioinfo.ut.ee/primer3-0.4.0) or Primer-BLAST (https://www.ncbi.nlm.nih.gov/tools/primer-blast/). In brief, frozen distal SI mucosa was homogenized, and total RNA was isolated with RNeasy Mini Kit (Qiagen, Hilden, Germany). The RNA concentration was measured using a NanoDrop 2000 spectrophotometer (Thermo Scientific), and 2 μg of isolated RNA was converted to single-stranded complementary DNA (cDNA) using High-Capacity cDNA Reverse Transcription Kit (Cat. No. 4368814, Applied Biosystems). For quantitative PCR, 10 μl of reaction mix consisting of 1.5 μl of cDNA, 200 nM of each primer, and 5 μl of 2× concentrated master mix (LightCycler 480 SYBR Green I Master, Cat. No. 04 887352001, Roche, Mannheim, Germany) were run under the following conditions: initial denaturation at 95°C for 10 min, 45 cycles of 15 s at 95°C, 30 s at 57°C, 30 s at 72°C for the signal detection. All samples were run in duplicates in 384-well reaction plates (LightCycler 480, Roche). The relative expression of target gene was calculated by 2^−ΔΔCT^ method and normalized to the expression level of housekeeping gene. The purity of PCR products was verified by melting curve analysis.

**Table 2:** List of primer sequences used for quantitative PCR (qPCR).

**Gene**	**Forward primer**	**Reverse primer**
*HPRT1*	5′–TATGGACAGGACTGAACGGC−3′	5′–ATCCAGCAGGTCAGCAAAGA−3′
*MYD88*	5′–GCAGCATCCCTTGGATGTCA−3′	5′–ATCCGACGGCACCTCTTTTC−3′
*LBP*	5′–CCCAAGGTCAATGATAAGTTGG−3′	5′–ATCTGGAGAACAGGGTCGTG−3′
*IL8*	5′–CTGTGAGGCTGCAGTTCTGG−3′	5′—CCAGGCAGACCTCTTTTCCAT−3′
*C3*	5′–ATCAAATCAGGCTCCGATGA−3′	5′–GGGCTTCTCTGCATTTGATG−3′
*MPO*	5′–CCCGAGTTGCTTTCCTCACT−3′	5'—AAGAAGGGGATGCAGTCACG−3′
*TLR4*	5′–TGGTGTCCCAGCACTTCATA−3′	5′–CAACTTCTGCAGGACGATGA−3'
*HIF1A*	5′–TGTGTTATCTGTCGCTTTGAGTC−3′	5′–TTTCGCTTTCTCTGAGCATTC−3′
*OCLN*	5′–GACGAGCTGGAGGAAGACTG−3′	5′–GTACTCCTGCAGGCCACTGT−3′
*CASP3*	5′–ATTGAGACGGACAGTGGGAC−3′	5′–GCTGCACAAAGTGACTGGAT−3′
*VEGF*	5′–ATGCGGATCAAACCTCACCA−3′	5′–TTTCGCTTTCTCTGAGCATTC−3′
*OLFM4*	5′–CGAATCCCAGTCGGTTTCCA−3′	5′–TGATTTCCAAGCGCTCCACT−3′

### Statistical Analysis

Repeated measurements (e.g., physical activity) were analyzed using the linear mixed effects model followed by group comparisons using the *lsmeans* package in the software package R (version 3.2.2). Binary outcomes, such as NEC incidence, were evaluated by multiple logistic regression models, and other continuous outcome measures (e.g., weight gain) were analyzed using linear models. Time-to-event data, such as motor skill acquisition, was analyzed using cox proportional hazard model. Ordered categorical outcomes (NEC severity score) were analyzed using a proportional odds logistic regression model. The abovementioned models were all adjusted for potential confounders, including birth weight, sex, litter, and life time. *Post-hoc* Dunnett's test with FF as the control group was performed to correct for the multiple comparisons for the four groups of 9-day pigs. The normality and homoscedasticity of the residuals and fitted values were performed for model validation, and data were transformed when required. Non-parametric analysis was applied when data could not be transformed properly. Data are presented as raw arithmetic means and SEM, unless otherwise stated. *P* < 0.05 was considered as statistically significant, and *P* ≤ 0.15 was discussed as a tendency to an effect.

## Results

### Clinical Outcomes and Organ Dimensions

**Figure 2:**
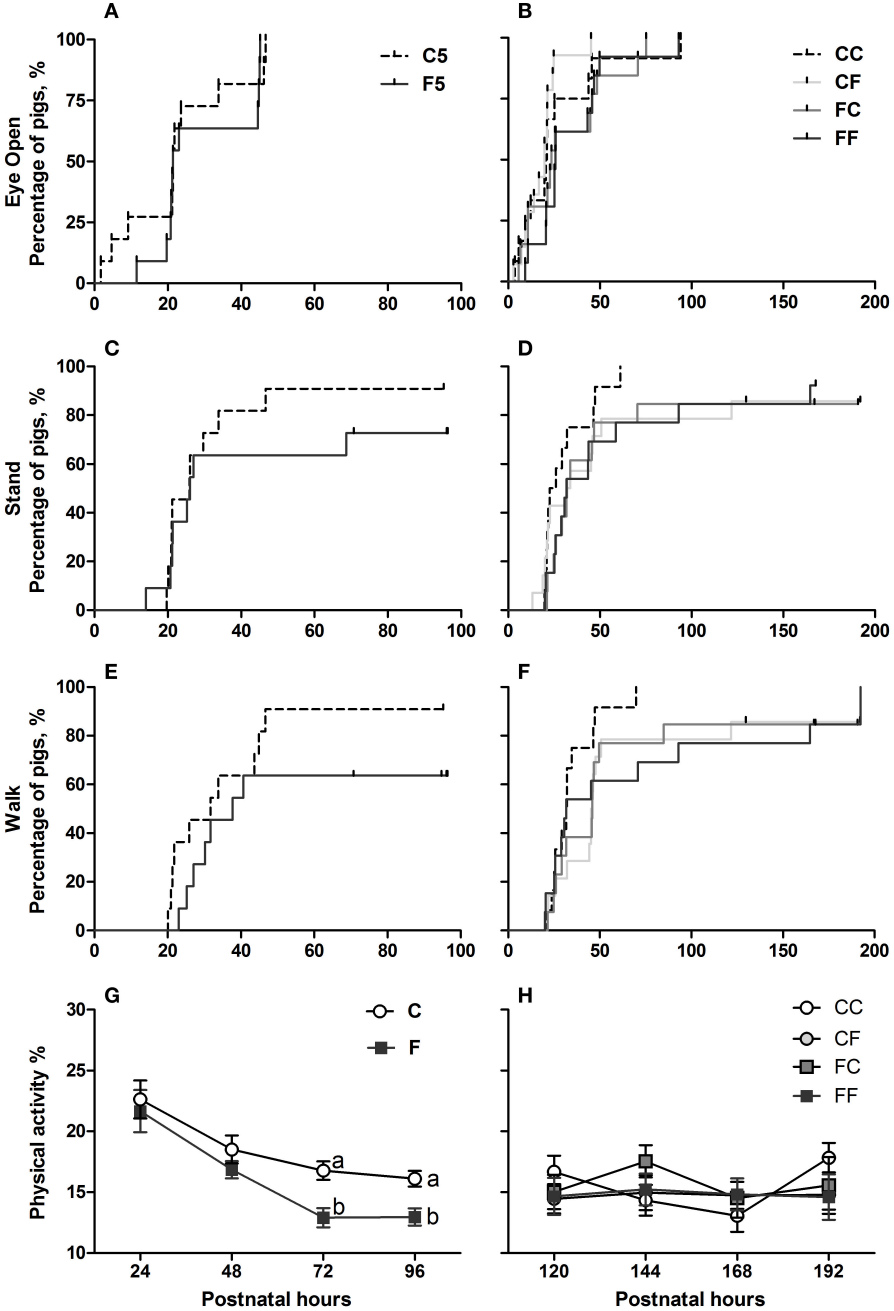
Time to acquisition of basic motor skills and home cage activity. Kaplan–Meier plots of time to first eyelid opening **(A,B)**, first stand **(C,D)**, and first walk **(E,F)** expressed as percentage of pigs. Home cage activity percentage over postnatal hours 24–48 **(G)** and 120–192 **(H)** expressed as means ± SEM. Means marked with different letters on the same day are significantly different, *P* < 0.05. *n* = 11 for C5 and F5 groups, *n* = 37 each for C and F groups, and *n* = 12–14 for CC, CF, FC, and FF groups. C, colostrum feeding for the first 4 days; CC, colostrum feeding until day 9; CF, 4 days colostrum feeding followed by formula feeding until day 9; F, formula feeding for the first 4 days; FC, 4 days formula feeding followed by colostrum feeding until day 9; FF, formula feeding until day 9.

Three pigs in the F5 group and none in the C5 group were euthanized before the scheduled date due to severe pain. Birth weights and lifetime did not differ between C5 and F5 groups, but C5 pigs had greater daily weight gain (*P* < 0.05, Table 3). In the 9-day pigs, birth weights, weights at euthanasia, daily weight gain, and lifetime did not differ among groups (Table 3). Pigs that received exclusive or initial colostrum feeding had less diarrhea on day 4 (C vs. F, *P* < 0.001) and day 8 (CC or CF vs. FF, respectively, both *P* < 0.05, Table 3). At day 5, NEC incidence was reduced in C5 pigs, especially concerning severe NEC lesions (NEC score ≥ 4, *P* < 0.05), and the lesions mainly differed between groups in the colon (*P* < 0.05, Table 3). In the 9-day pigs, the incidences of both overall and severe NEC were similar among groups (Table 3). Systemic infection indicated by bacterial counts in bone marrow or CSF did not differ significantly among groups (Table 3). *Enterococcus* and *Staphylococcus* were the two genera identified in bone marrow tissues. Motor skill acquisition monitored by the time to first eye opening, standing, and walking did not differ among groups (Figure 2). The proportion of physical activity (a marker of well-being) was greater in colostrum- than formula-fed pigs on days 3 and 4 (*P* < 0.05 on both days), but was similar among groups for the remaining days (Figure 2).

**Table 3:** Clinical outcomes and organ dimensions^a^.

	**C5**	**F5**	***P*_**1**_**	**CC**	**CF**	**FC**	**FF**	***P*_**2**_**
*N*	10–11	10–11		11–12	13–14	11–13	13	
Birth weight (g)	1,104 ± 65	1,086 ± 91	0.684	1,135 ± 51	1,032 ± 80	976 ± 88	986 ± 84	0.643
Kill weight (g)	1,190 ± 72	1,119 ± 94	<0.05	1,437 ± 59	1,251 ± 103	1,120 ± 107	1,206 ± 108	0.757
Weight gain (g/day)	21 ± 3	8 ± 3	<0.05	33 ± 4	28 ± 4	25 ± 4	27 ± 4	0.789
Life time (h)	96 ± 0.2	90 ± 3.0	0.355	193 ± 0.5	188 ± 4.5	189 ± 2.4	190 ± 2.1	0.524
Diarrhea incidence on day 4, *n* (%)^b^	6 (16)	18 (49)	<0.001	–	–	–	–	–
Diarrhea incidence on day 8, *n* (%)	–	–	–	3 (25)^**^	6 (43)^*^	9 (69)	11 (85)	<0.001
NEC incidence, *n* (%)	5 (45)	8 (73)	0.097	8 (67)	10 (71)	8 (62)	10 (77)	0.798
Severe NEC incidence, *n* (%)	3 (27)	7 (64)	<0.05	2 (17)	3 (21)	2 (15)	2 (15)	0.988
SI lesion score	2.00 ± 0.43	2.36 ± 0.51	0.216	1.83 ± 0.34	2.07 ± 0.27	1.31 ± 0.17	1.38 ± 0.24	0.165
Colon lesion score	1.64 ± 0.34	3.00 ± 0.49	<0.01	2.50 ± 0.36	2.50 ± 0.27	2.62 ± 0.33	2.85 ± 0.22	0.780
Bacteria in bone marrow/CSF, *n* (%)	6 (55)	7 (78)	0.290	5 (42)	7 (54)	9 (69)	10 (83)	0.288
Bacterial load in bone marrow, cfu/g, median (IQR) × 10^4^	0.3 (0–0.5)	1.3 (0–1.9)	0.239	0 (0–4.7)	1.0 (0–2.6)	0.3 (0–11)	0.6 (0.2–4.4)	0.695
Organ dimensions, relative to kill weight									
Stomach (g/kg)	5.8 ± 0.4	6.6 ± 0.5	<0.05	7.6 ± 1.0	6.0 ± 0.3	8.7 ± 1.5	6.5 ± 0.3	0.225
Stomach content (g/kg)	12.5 ± 1.7	11.1 ± 2.1	0.986	14.1 ± 2.4	15.2 ± 1.2	9.8 ± 2.4^c^	16.0 ± 2.2	0.084
Small intestine (g/kg)	29.2 ± 1.1	27.7 ± 1.0	0.466	34.7 ± 1.6	34.7 ± 1.5	31.3 ± 1.0	32.2 ± 1.2	0.167
Small intestinal length (cm/kg)	276 ± 11	286 ± 13	0.404	267 ± 12	298 ± 17	300 ± 20	318 ± 21	0.360
Colon (g/kg)	12.8 ± 0.8	13.3 ± 1.1	0.221	16.1 ± 1.1^*^	21.6 ± 1.6	16.5 ± 1.1^d^	20.4 ± 1.5	<0.01
Liver (g/kg)	22.5 ± 0.8	26.1 ± 1.4	<0.05	25.2 ± 1.2	25.8 ± 0.8	26.4 ± 1.8	27.6 ± 2.0	0.948
Spleen (g/kg)	1.85 ± 0.13	2.67 ± 0.36	<0.001	2.96 ± 0.22	2.68 ± 0.18	2.56 ± 0.17	2.71 ± 0.16	0.342
Heart (g/kg)	7.3 ± 0.2	7.3 ± 0.2	0.412	6.9 ± 0.3	7.0 ± 0.2	7.3 ± 0.2	6.8 ± 0.2	0.251
Lungs (g/kg)	23.6 ± 1.7	25.5 ± 2.2	0.286	26.5 ± 1.9	26.1 ± 0.9	24.5 ± 1.0	24.2 ± 1.2	0.627
Kidneys (g/kg)	9.1 ± 0.9	9.6 ± 0.6	0.961	8.8 ± 0.4	8.7 ± 0.3	9.2 ± 0.6	9.5 ± 0.5	0.499
Adrenal glands (g/kg)	0.19 ± 0.01	0.19 ± 0.01	0.561	0.17 ± 0.02	0.18 ± 0.01	0.16 ± 0.02	0.18 ± 0.02	0.705

a Values are means ± SEMs unless otherwise noted; P_1_ indicates the P value comparing the two 5-day groups, i.e., C5 and F5; P_2_ indicates the P-value comparing the four 9-day groups, i.e., CC, CF, FC, and FF; The post-hoc comparison between the respective three groups and the control group (FF) was performed by Dunnett's test, and significance was indicated by superscript asterisks:

* *P < 0.05*,

** *P < 0.01*.

b *Values are diarrhea incidence on day 4 in all pigs fed colostrum or formula. N = 37 in each group*.

c *FC vs. FF, P = 0.067*.

d *FC vs. FF, P = 0.078*.

*C5, colostrum feeding until day 5; CC, colostrum feeding until day 9; CF, 4 days colostrum feeding followed by formula feeding until day 9; CSF, cerebrospinal fluid; F5, formula feeding until day 5; FC, 4 days formula feeding followed by colostrum feeding until day 9; FF, formula feeding until day 9; NEC, necrotizing enterocolitis*.

On day 5, the relative weights of the stomach, liver, and spleen were all lower in the C5 vs. F5 pigs (all *P* < 0.05, Table 3), while on day 9, these and other organ weights were similar among groups. However, at this time point, stomach content weight tended to be less in FC vs. FF pigs (*P* = 0.067, Table 3), and likewise, the relative weight of the colon (including its contents) tended to be lower in the pigs fed colostrum exclusively or for the last days, relative to pigs fed exclusive formula (*P* < 0.08 for CC vs. FF and FC vs. FF, Table 3).

### Blood Biochemistry

Blood biochemistry parameters (Table 4) showed that, despite similar protein intake, C5 pigs had lower albumin concentrations than F5 pigs on day 5 (*P* < 0.05), although these were similar among groups on day 9. In terms of liver functions, γ*-*glutamyltransferase was elevated, and total bilirubin was lowered in C5 vs. F5 pigs (both *P* < 0.05) but similar among groups on day 9. Conversely, cholesterol levels were highest in 9-day-old pigs fed colostrum exclusively or for the last 4 days (CC or FC vs. FF, respectively, both *P* < 0.05), while there was only a tendency to an effect at 5 days (*P* = 0.122 for C5 vs. F5). The creatinine concentration was similar on day 5 but decreased significantly thereafter in the FF group compared with the CC group (*P* < 0.05). The last 4 days of colostrum feeding in the FC group tended to increase creatinine levels compared with FF pigs (*P* = 0.094). For blood glucose, iron, and calcium, the pigs fed colostrum exclusively or for the last 4 days (CC, FC), but not for the first 4 days (CF), had lower values than FF pigs (CC or FC vs. FF, respectively, both *P* < 0.1). For magnesium and ionized phosphate levels, colostrum-fed pigs had higher values on day 5 (C5 vs. F5, both *P* < 0.01). On day 9, ionized phosphate levels were increased by colostrum fed exclusively or for the last days (CC or FC vs. FF, respectively, both *P* < 0.001), and the increase was to a less degree for magnesium levels, both *P* < 0.1, Table 3).

**Table 4:** Blood biochemistry parameters^a^.

	**C5**	**F5**	***P*_**1**_**	**CC**	**CF**	**FC**	**FF**	***P*_**2**_**
*N*	11	11		12	14	13	13	
Total protein (g/L)	26.1 ± 0.3	26.5 ± 0.9	0.132	28.4 ± 0.9	27.5 ± 0.7	29.3 ± 1.6	28.2 ± 0.8	0.692
Albumin (g/L)	9.9 ± 0.3	10.6 ± 0.4	< 0.05	12.7 ± 0.6	12.2 ± 0.4	13.6 ± 1.0	13.2 ± 0.4	0.527
ALP (U/L)	*2, 506*±328	*2, 145*±311	0.175	*1, 447*±102	*1, 883*±77	*1, 698*±285	*1, 700*±180	0.190
ALT (U/L)	16.0 ± 0.9	16.1 ± 0.6	0.451	16.2 ± 0.8	16.1 ± 0.7	17.5 ± 1.2	16.9 ± 0.6	0.549
AST (U/L)	31.5 ± 9.3	34.0 ± 10.0	0.785	20.7 ± 1.6	33.5 ± 5.4	57.9 ± 12.4	27.6 ± 1.9	< 0.05
GGT (U/L)	46.9 ± 5.9	26.3 ± 3.5	< 0.05	34.8 ± 3.5	33.9 ± 3.0	26.2 ± 2.5	32.5 ± 3.9	0.364
Total bilirubin (μmol/L)	1.7 ± 0.4	3.2 ± 0.6	< 0.05	1.1 ± 0.3	1.1 ± 0.2	2.1 ± 0.4	1.5 ± 0.3	0.350
Cholesterol (mmol/L)	2.2 ± 0.2	1.8 ± 0.2	0.122	2.4 ± 0.2^**^	2.0 ± 0.1	2.2 ± 0.2^*^	1.6 ± 0.1	< 0.05
Creatinine (μmol/L)	48.8 ± 2.4	47.7 ± 1.5	0.194	48.0 ± 1.8^*^	43.1 ± 1.7	44.8 ± 2.5^b^	38.9 ± 2.4	0.054
Creatine kinase (U/L)	81 ± 11	80 ± 11	0.797	71 ± 5	117 ± 21	170 ± 32	96 ± 6	< 0.05
BUN (mmol/L)	5.0 ± 0.7	5.8 ± 0.6	0.893	10.0 ± 1.1	10.3 ± 0.9	9.5 ± 1.0	10.4 ± 0.9	0.736
Lactate (mmol/L)	1.4 ± 0.3	1.5 ± 0.2	0.585	1.9 ± 0.3	1.6 ± 0.2	2.2 ± 0.3	2.1 ± 0.5	0.572
Glucose (mmol/L)	2.2 ± 0.3	2.5 ± 0.3	0.580	2.8 ± 0.2^c^	3.4 ± 0.3	3.0 ± 0.4	3.8 ± 0.3	0.114
Iron (μmol/L)	4.8 ± 1.0	4.0 ± 0.7	0.205	3.2 ± 0.6	4.0 ± 0.5	2.9 ± 0.5	3.9 ± 0.7	0.140
Calcium (mmol/L)	2.5 ± 0.0	2.5 ± 0.1	0.723	2.37 ± 0.03^*^	2.54 ± 0.03	2.39 ± 0.08^d^	2.52 ± 0.04	< 0.01
Magnesium (mmol/L)	0.87 ± 0.05	0.71 ± 0.02	< 0.001	0.91 ± 0.05^**^	0.71 ± 0.02	0.83 ± 0.03^e^	0.73 ± 0.02	< 0.001
Ionized phosphate (mmol/L)	1.5 ± 0.0	1.3 ± 0.1	< 0.01	1.7 ± 0.1^***^	1.2 ± 0.1	1.9 ± 0.1^***^	1.1 ± 0.1	< 0.0001
Sodium (mmol/L)	144 ± 1	146 ± 1	0.983	140 ± 1	143 ± 1	145 ± 3	144 ± 1	0.560
Potassium (mmol/L)	4.1 ± 0.1	4.0 ± 0.2	0.342	4.0 ± 0.2	3.8 ± 0.1	4.1 ± 0.2	4.0 ± 0.2	0.655

a Values are means ± SEMs; P_1_ indicates the P-value comparing the two 5-day groups, i.e., C5 and F5; P_2_ indicates the P-value comparing the four 9-day groups, i.e., CC, CF, FC, and FF; the post-hoc comparison between the respective three groups and the control group (FF) was performed by Dunnett's test, and significance was indicated by superscript asterisks:

* *P < 0.05*,

** *P < 0.01*,

*** *P < 0.001*.

b *FC vs. FF, P = 0.094*.

c *CC vs. FF, P = 0.078*.

d *FC vs. FF, P = 0.099*.

e *FC vs. FF, P = 0.091*.

*ALP, alkaline phosphatase; ALT, alanine aminotransferase; AST, aspartate aminotransferase; GGT, gamma-glutamyltransferase; BUN, blood urea nitrogen; C5, colostrum feeding until day 5; CC, colostrum feeding until day 9; CF, 4 days colostrum feeding followed by formula feeding until day 9; CSF, cerebrospinal fluid; F5, formula feeding until day 5; FC, 4 days formula feeding followed by colostrum feeding until day 9; FF, formula feeding until day 9*.

### Blood Cell Count, Neutrophil Phagocytosis, T-Cell Profiling, and Plasma Mediators

Blood cell count results are shown in Table 5, and a few parameters differed among groups at both time points. On day 5, the neutrophil counts and proportions were higher in the C group, whereas the proportion of lymphocytes and large unstained cells (reflecting activated lymphocytes and peroxidase-negative cells) were less than in the F group (all *P* < 0.05). Interestingly, both neutrophil count and proportion increased on day 9 in all pigs, but the increment was to a lesser degree in CF pigs (CF vs. FF, both *P* < 0.05). Accordingly, the proportions of lymphocyte and monocyte decreased from day 5 to 9, in general, but the decline in lymphocyte proportion was smaller and the monocyte proportion did not decrease in the CF group (CF vs. FF, both *P* < 0.05). The proportion of eosinophils was similar on day 5 between the groups and tended to be lower in CC vs. FF pigs on day 9 (*P* = 0.065).

**Table 5:** Blood cell count parameters^a^.

	**C**	**F**	***P*_**1**_**	**CC**	**CF**	**FC**	**FF**	***P_**2**_***
*N*	24	24		9	10	5	8	
Total leukocyte count (10^9^/L)	2.70 ± 0.18	2.44 ± 0.28	0.192	8.35 ± 1.92	6.22 ± 0.89^*^	6.22 ± 0.54	9.06 ± 1.86	0.105
Total erythrocyte count (10^9^/L)	3.90 ± 0.09	3.71 ± 0.11	0.283	2.69 ± 0.46	3.85 ± 0.42	3.17 ± 0.39	3.29 ± 0.17	0.403
Hemoglobin	4.95 ± 0.10	4.55 ± 0.23	0.162	3.19 ± 0.56	4.58 ± 0.42	3.76 ± 0.42	3.89 ± 0.22	0.354
Hematocrit	0.28 ± 0.01	0.27 ± 0.01	0.490	0.19 ± 0.03	0.26 ± 0.02	0.22 ± 0.03	0.23 ± 0.01	0.351
MCV	72.6 ± 0.7	73.4 ± 0.8	0.583	69.4 ± 1.0	69.4 ± 0.84	70.0 ± 1.0	69.5 ± 0.8	0.993
MCHC	17.5 ± 0.1	16.7 ± 0.7	0.279	16.3 ± 0.8	17.3 ± 0.1	16.8 ± 0.4	17.0 ± 0.1	0.661
Platelets	101 ± 21	86 ± 18	0.685	464 ± 96	496 ± 53	557 ± 97	426 ± 56	0.497
MPV	13.7 ± 0.9	15.1 ± 0.7	0.280	12.3 ± 1.1	12.3 ± 0.8	15.3 ± 2.8	14.7 ± 1.3	0.980
MPC	236 ± 2	236 ± 2	0.928	242 ± 6	245 ± 4	236 ± 8	244 ± 6	0.155
Neutrophil count (10^9^/L)	1.43 ± 0.13	1.17 ± 0.23	< 0.05	5.97 ± 1.65	3.89 ± 0.79^*^	3.97 ± 0.48	6.86 ± 1.83	< 0.05
Neutrophil proportion (%)	52.3 ± 2.1	43.8 ± 2.8	< 0.01	65.1 ± 4.0	57.7 ± 5.2^*^	64.0 ± 5.7	69.9 ± 4.8	< 0.05
Lymphocyte count (10^9^/L)	1.08 ± 0.07	1.06 ± 0.07	0.943	1.88 ± 0.23	1.93 ± 0.16	2.01 ± 0.38	1.76 ± 0.20	0.899
Lymphocyte proportion (%)	41.3 ± 2.0	47.8 ± 2.7	< 0.05	28.7 ± 3.7	35.4 ± 4.8^*^	32.0 ± 5.5	25.2 ± 4.5	0.065
Monocyte count (10^9^/L)	0.12 ± 0.02	0.12 ± 0.02	0.830	0.29 ± 0.09	0.27 ± 0.04	0.14 ± 0.02	0.20 ± 0.04	0.729
Monocyte proportion (%)	4.3 ± 0.5	4.9 ± 0.5	0.228	3.3 ± 0.4	4.5 ± 0.5^*^	2.2 ± 0.5	2.5 ± 0.6	< 0.05
Eosinophil count (10^8^/L)	0.11 ± 0.02	0.09 ± 0.02	0.249	0.36 ± 0.11	0.32 ± 0.04	0.42 ± 0.10	0.95 ± 0.50	0.262
Eosinophil proportion (%)	0.4 ± 0.1	0.4 ± 0.1	0.718	0.4 ± 0.1^b^	0.6 ± 0.1	0.7 ± 0.2	0.9 ± 0.3	0.106
Basophil count (10^8^/L)	0.04 ± 0.01	0.05 ± 0.01	0.717	0.08 ± 0.03	0.09 ± 0.01	0.14 ± 0.06	0.11 ± 0.04	0.975
Basophil proportion (%)	0.2 ± 0.02	0.2 ± 0.03	0.546	0.2 ± 0.05	0.2 ± 0.02	0.2 ± 0.08	0.2 ± 0.04	0.769
LUC count (10^8^/L)	0.45 ± 0.09	0.72 ± 0.15	0.066	1.56 ± 0.38	0.94 ± 0.15	0.54 ± 0.14	1.29 ± 0.41	0.421
LUC proportion (%)	1.5 ± 0.3	2.8 ± 0.3	< 0.001	2.2 ± 0.5	1.7 ± 0.3	0.9 ± 0.02	1.5 ± 0.3	0.355

a Values are means ± SEMs; P_1_ indicates the P-value comparing the two groups fed colostrum or formula on day 5, i.e., C and F; P_2_ indicates the P-value comparing the four 9-day groups, i.e., CC, CF, FC, and FF; the post-hoc comparison between the respective three groups and the control group (FF) was performed by Dunnett's test and significance was indicated by superscript asterisks:

* *P < 0.05*.

b *CC vs. FF, P = 0.063*.

*C, colostrum feeding; CC, colostrum feeding until day 9; CF, 4 days colostrum feeding followed by formula feeding until day 9; CSF, cerebrospinal fluid; F, formula feeding; FC, 4 days formula feeding followed by colostrum feeding until day 9; FF, formula feeding until day 9; LUC, large unstained cells; MCHC, mean corpuscular hemoglobin concentration; MCV, mean corpuscular volume; MPC, mean platelet component; MPV, mean platelet volume*.

**Figure 3:**
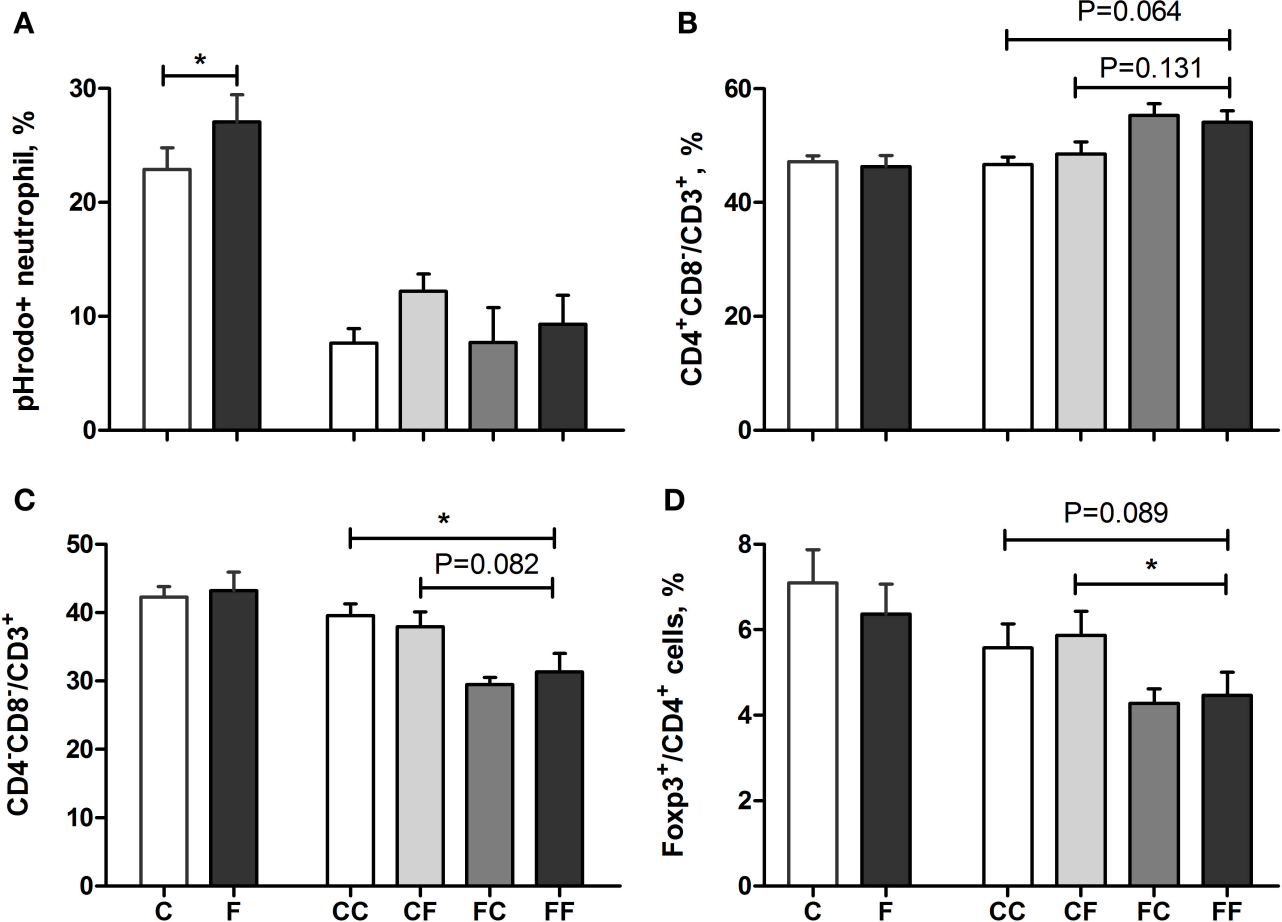
Systemic immunity, including neutrophil function and blood T-cell subsets. Neutrophil phagocytic rate **(A)**, T_h_ frequency **(B)**, DN frequency **(C)**, and T_reg_ frequency **(D)**. *n* = 21–27 for C and F groups and *n* = 4–8 for CC, CF, FC, and FF groups. Values (means ± SEM) designated with asterisks are significantly different, *P < 0.05; T_h_, helper T cells. DN, double-negative T cells; T_reg_, regulatory T cells; C, colostrum feeding for the first 4 days; CC, colostrum feeding until day 9; CF, 4 days colostrum feeding followed by formula feeding until day 9; F, formula feeding for the first 4 days; FC, 4 days formula feeding followed by colostrum feeding until day 9; FF, formula feeding until day 9.

The proportion of phagocytic neutrophils (pHrodo^+^ neutrophils) was lower in the colostrum-fed pigs compared with formula-fed pigs (C vs. F, *P* < 0.05) on day 5 and decreased to ~10% across groups by day 9 (Figure 3A). The frequency of blood T cells (CD3^+^ lymphocytes) in the lymphocyte population did not differ among groups on both days (59 ± 2% in both 5-day and 9-day pigs). The frequency of cytotoxic T cells (T_c_, CD3^+^CD4^−^CD8^+^) in the T-cell population was similar among groups on both days (9.9 ± 0.7% on day 5 and 13.0 ± 0.5% on day 9). The frequency of helper T cells (T_h_, CD3^+^CD4^+^CD8^−^) in the T-cell population was similar between the C and F groups, and on day 9, the frequency tended to be lower in the CC and CF groups compared with the FF group (*P* = 0.064 and 0.131, respectively, Figure 3B). Accordingly, the frequency of DN (CD3^+^CD4^−^CD8^−^) in total T cell was higher or tended to be higher in the CC and CF groups compared with the FF group (*P* = 0.082 and <0.05, respectively, Figure 3C). The frequency of T_reg_ cells (CD3^+^CD4^+^Foxp3^+^) in the T_h_ population did not differ on day 5, but was higher in CC and CF pigs, relative to FF pigs with either a statistical significance or a tendency (*P* = 0.089 and <0.05, respectively, Figure 3D). The ratio of helper/cytotoxic T cells was similar for both diet and age (6.7 ± 0.7 on day 5 and 4.1 ± 0.2 on day 9 across groups).

Collectively, relative to initial formula feeding (pooled FF and FC pigs), initial colostrum feeding (pooled CC and CF pigs) decreased the frequency of T_h_ (CC + CF vs. FC + FF, 48 ± 1 vs. 55 ± 1%) and increased the frequencies of DN (39 ± 1 vs. 31 ± 2%) and T_reg_ (6 ± 0.3 vs. 4 ± 0.3%) in the T-cell population (all *P* < 0.05). Finally, plasma IL1-β was below the detection limit, and the levels of C-reactive protein and IL-6 were similar among groups on day 5 (2.69 ± 0.52 mg/L and 0.53 ± 0.10 ng/ml across groups) and on day 9 (5.09 ± 0.43 mg/L and 0.37 ± 0.02 ng/ml across groups).

### Gut Histology, *in vivo* Intestinal Function, and Brush Border Enzyme Activities

**Figure 4:**
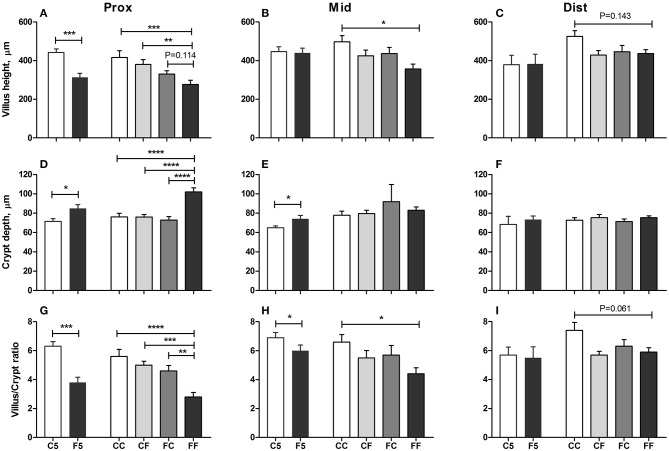
Mucosal morphology. Villus heights in the proximal, middle, and distal small intestine **(A–C)**, crypt depth in the proximal, middle, and distal small intestine **(D–F)**, villus/crypt ratio in the proximal, middle, and distal small intestine **(G–I)**. Values (means ± SEM) designated with asterisks are significantly different, *P < 0.05, **P < 0.01, ***P < 0.001, ****P < 0.0001. *n* = 11 for C5 and F5 groups and *n* = 12–14 for CC, CF, FC, and FF groups. C5, colostrum feeding until day 5; CC, colostrum feeding until day 9; CF, 4 days colostrum feeding followed by formula feeding until day 9; F5, formula feeding until day 5; FC, 4 days formula feeding followed by colostrum feeding until day 9; FF, formula feeding until day 9.

**Figure 5:**
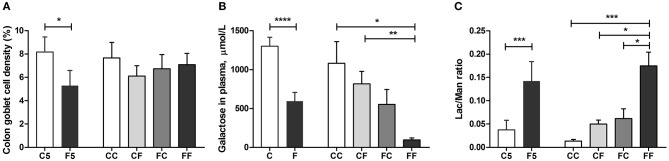
Goblet cell density and *in vivo* intestinal functions. Goblet cell density in the colon **(A)**, intestinal hexose absorptive capacity on days 4 and 8 **(B)**, and intestinal permeability measure before euthanasia **(C)**. Values (means ± SEM) designated with asterisks are significantly different, *P < 0.05, **P < 0.01, ***P < 0.001, ****P < 0.0001. *n* = 8–11 for C5 and F5 groups, *n* = 33 each for C and F groups, and *n* = 7–14 for CC, CF, FC, and FF groups. C, colostrum feeding for the first 4 days; C5, colostrum feeding until day 5; CC, colostrum feeding until day 9; CF, 4 days colostrum feeding followed by formula feeding until day 9; F, formula feeding for the first 4 days; F5, formula feeding until day 5; FC, 4 days formula feeding followed by colostrum feeding until day 9; FF, formula feeding until day 9.

Colostrum feeding increased the villus height relative to formula feeding in the proximal region of the SI on both day 5 and 9 (both *P* < 0.001, Figure 4A), and in the middle SI, the increment was only seen on day 9 (*P* < 0.05, Figure 4B). In the proximal SI, both CF and FC groups had increased villus height either significantly or with a tendency, relative to the FF group (*P* < 0.001 and *P* = 0.114, respectively, Figure 4A). When it comes to the crypt depth and villus/crypt ratio, any colostrum feeding (i.e., C5, CC, CF, and FC) could decrease the depth or increase the ratio, relative to formula feeding for either 4 days (i.e., F5) or 8 days (i.e., FF, all *P* < 0.05, Figures 4D,G). In the middle region, crypt depth was decreased in the colostrum-fed pigs on day 5 (C5 vs. F5, *P* < 0.05), and villus/crypt ratio was increased in colostrum-fed pigs both on day 5 (C5 vs. F5, *P* < 0.05) and on day 9 (CC vs. FF, *P* < 0.05, Figures 4E,H). In the distal region, only tendencies of increased villus height and villus/crypt ratio were observed in the CC vs. FF groups (*P* = 0.143 and *P* = 0.061, respectively, Figures 4C,I), and no difference was observed on crypt depth in this region (Figure 4F). Mucin-containing goblet cell density in the colon was also increased by colostrum feeding on day 5 (C5 vs. F5, *P* < 0.05), and on day 9, formula-fed pigs start to have similar density to the colostrum-fed pigs (Figure 5A).

**Figure 6:**
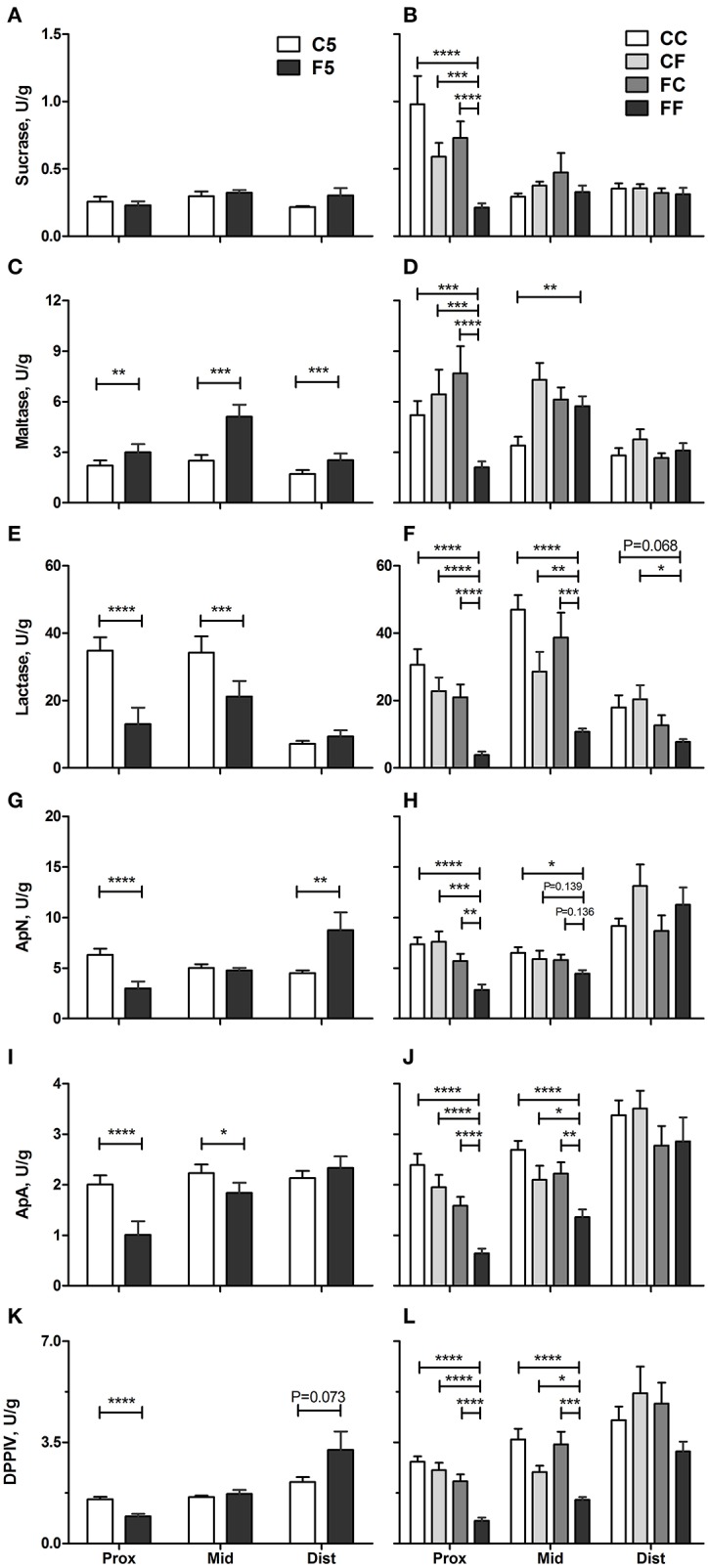
Brush border enzyme activities. Sucrase **(A,B)**, maltase **(C,D)**, lactase **(E,F)**, ApN **(G,H)**, ApA **(I,J)**, and DPPIV **(K,L)** in the proximal, middle, and distal small intestine. Values (means ± SEM) designated with asterisks are significantly different, *P < 0.05, **P < 0.01, ***P < 0.001, ****P < 0.0001. *n* = 11 each for C5 and F5 groups, and *n* = 12–14 for CC, CF, FC, and FF groups. ApA, aminopeptidase A; ApN, aminopeptidase N; C5, colostrum feeding until day 5; CC, colostrum feeding until day 9; CF, 4 days colostrum feeding followed by formula feeding until day 9; DPPIV, dipeptidylpeptidase IV; F5, formula feeding until day 5; FC, 4 days formula feeding followed by colostrum feeding until day 9; FF, formula feeding until day 9.

*In vivo* intestinal functions such as hexose uptake and intestinal permeability were all improved in the colostrum-fed compared with the formula-fed group on day 5 (all *P* < 0.001, Figures 5B,C). On day 9, hexose absorption was increased in CC and CF groups, and the permeability was decreased in CC, CF, and FC groups, relative to the FF group (all *P* < 0.05, Figures 5B,C). Compared with the F5 group, the activity of brush border sucrose was similar, while maltase was lower in the C5 group in all SI regions (all *P* < 0.01, Figures 6A,C), whereas the other enzyme activities were all increased in the C5 group in the proximal region and in one of the middle and distal regions (all *P* < 0.05, Figures 6E,G,I,K). On day 9, all colostrum-feeding groups, i.e., CC, CF, and FC, had higher enzyme activities than the FF group (all *P* < 0.01, Figures 6B,D,F,H,J,L). The activities of lactase, ApA, and DPPIV in the middle SI were also increased by any colostrum feeding (CC, CF, and FC vs. FF, respectively, all *P* < 0.05, Figures 6B,F,J,L). The maltase activity was decreased and ApN activity was increased in CC vs. FF groups in the middle region (both *P* < 0.05, Figures 6D,H). Distal enzyme activities were not influenced by feeding in general on day 9, except for the lactase activity being marginally higher in CC and CF groups compared with FF group (*P* = 0.068 and *P* < 0.05, respectively, Figure 6F). Collectively, the results indicate that any colostrum feeding (provided to CC, CF, or FC pigs) reduced the damaging effects of a period of formula feeding (provided to FF pigs) in the intestinal structure, function, and integrity.

### Gene Expression Related to Inflammation, Proliferation, and Tight Junctions

**Figure 7:**
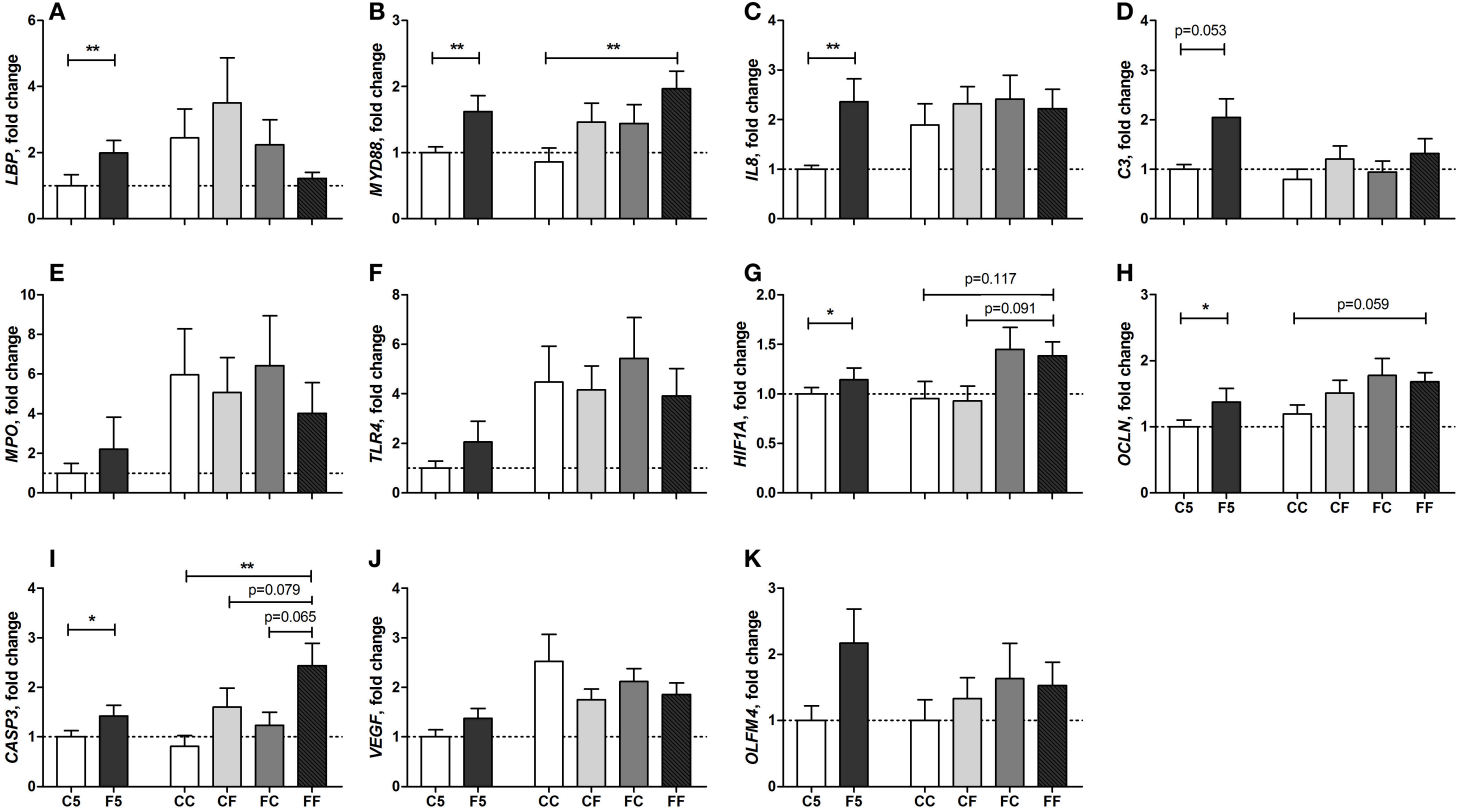
Fold change of intestinal gene expression levels relative to the C5 group. *LBP* **(A)**, *MYD88* **(B)**, *IL8* **(C)**, *C3* **(D)**, *MPO* **(E)**, *TLR4* **(F)**, *HIF1A* **(G)**, *OCLN* **(H)**, *CASP3* **(I)**, *VEGF* **(J)**, *OLFM4* **(K)** in the distal small intestine. Values (means ± SEM) designated with asterisks are significantly different, ^*^P < 0.05, ^**^P < 0.01. *n* = 8 each for C5 and F5 groups, and *n* = 9–13 for CC, CF, FC, and FF groups. C5, colostrum feeding until day 5; CC, colostrum feeding until day 9; CF, 4 days colostrum feeding followed by formula feeding until day 9; F5, formula feeding until day 5; FC, 4 days formula feeding followed by colostrum feeding until day 9; FF, formula feeding until day 9.

The genes related to gut innate immune response, such as lipopolysaccharide (LPS) binding protein (*LBP*), myeloid differentiation primary response 88 (*MYD88*), interleukin-8 (*IL8*), and complement C3 (*C3*) were all upregulated in F5 compared with C5 pigs on day 5 (all *P* < 0.01, except for *C3, P* = 0.053, Figures 7A–D). The genes related to tissue hypoxia, integrity, and apoptosis, namely, hypoxia inducible factor 1 subunit alpha (*HIF1A*), occludin (*OCLN*), and caspase-3 (*CASP3*), were also upregulated in the F5 group (all *P* < 0.05, Figures 7G–I). The genes related to immune defense, myeloperoxidase (MPO) and toll-like receptor 4 (TLR4), angiogenesis, vascular endothelial growth factor (*VEGF*), and an intestinal stem cell marker, olfactomedin 4 (*OLFM4*), did not differ between C5 and F5 groups (Figures 7E,F,J,K). On day 9, the expression of *MYD88* (*P* < 0.01, Figure 7B), *HIF1A* (*P* = 0.117, Figure 7G), *OCLN* (*P* = 0.059, Figure 7H), and *CASP3* (*P* < 0.01, Figure 7I) remained higher in the formula-fed pigs (FF group), relative to pigs fed with only colostrum (CC group). Feeding colostrum before or after a period of formula feeding (CF or FC) did not modulate the expression of analyzed genes except for some tendencies in *HIF1A* (CF vs. FF, *P* = 0.091, Figure 7G) and *CASP3* (CF and FC vs. FF, *P* = 0.079 and 0.065, respectively, Figure 7I). Collectively, proinflammatory response was mainly dampened by colostrum feeding during the first 5 days, whereas hypoxia and apoptosis response was downregulated by initial or any period of colostrum feeding.

## Discussion

We have previously shown the effects of colostrum feeding on systemic immunity, gut function and immunity, and NEC protection in this preterm pig model. Systemically, early colostrum feeding prevented septic shock and ameliorated neuroinflammation in preterm piglets that received intra-arterial *Staphylococcus epidermidis* (15). In the gut, piglets fed with colostrum as minimal enteral nutrition (12), full enteral nutrition (16), or supplementation to donor milk (17, 19) showed improved digestive functions, integrity, anti-inflammatory responses, and NEC resistance. Several clinical trials are being performed in very preterm infants to document this for its clinical application (14, 27, 28). A pilot study has demonstrated the feasibility and tolerability of initial colostrum feeding (27, 28), which has led to an ongoing randomized clinical trial powered for TFF as the primary outcome in very preterm infants (ClinicalTrials.gov Identifier: NCT03085277). How colostrum feeding before or after suboptimal formula feeding protects or restores formula-induced intestinal impairment still remains unclear. Similar to previous short-term studies (5 days), our results show that colostrum feeding improved weight gain and protected against NEC and diarrhea. The intestinal structure, digestive and absorptive functions, and barrier function were all improved by colostrum feeding, accompanied by lower expression of genes related to innate immune activation, apoptosis, and hypoxia in the gut tissue. Meanwhile, colostrum feeding increased total blood neutrophil count/proportion, indicating the maturational effect of colostrum in innate immunity. Newly recruited neutrophils may not be fully functional, explaining the higher portion of non-phagocytic neutrophils in colostrum- vs. formula-fed pigs.

In a longer term study when following the pigs for 9 days, diet-dependent NEC sensitivity decreased with age and weight gain increased to the similar levels in both colostrum- and formula-fed pigs, whereas the diarrhea incidence remained higher in the formula-fed pigs. The intestinal structure, functions, and integrity remained superior in the colostrum pigs, and apoptosis and hypoxia-related genes were still expressed at a lower level. Colostrum feeding for 8 days decreased the frequency of T_h_ and increased the frequencies of double-genitive T_c_ and T_reg_. Initial 4 days colostrum feeding was able to prevent later formula-induced diarrhea and gut structural and functional impairment. In addition, the frequencies of T_h_, double-genitive T_c_ and T_reg_, and apoptosis/hypoxia-related genes were similarly regulated by first 4 days colostrum feeding as exclusive colostrum feeding. Later 4 days colostrum feeding could restore formula-induced gut impairment and the upregulation of an apoptosis gene.

In this study, NEC lesions were more subclinical and primarily contained to the colon region, similar to our previous studies using gradual advancement of enteral feeding (12). More severe NEC cases were observed in formula-fed pigs on day 5, but the severity decreased over time in the 9-day-old pigs. This differed from our earlier studies where formula feeding increased NEC severity relative to colostrum both on day 5 (12) and in longer term studies [57% severe NEC on day 11 (18)]. Maldigestion of maltodextrin, the common carbohydrate source in preterm formula (29), is considered to induce NEC, at least in preterm pigs (30). Maltodextrin was used in the current study to induce NEC, but the concentration was low to match the carbohydrate level in colostrum (27 g/L), which may partly explain the lower NEC severity in formula-fed pigs compared with the previous study [84 g/L (18)]. Moreover, colon barrier function indicated by the density of mucin-containing goblet cells was initially reduced by formula feeding and restored over time, which may also be related to the decline of NEC severity over time. Genes related to immune defense (TLR4, MPO), barrier function (OCLN), and angiogenesis (VEGF) were generally higher on day 9 than on day 5 disregarding feeding. In addition, gut microbiota also play important role in NEC development. A systemic review reported microbial dysbiosis before NEC onset as increased *Proteobacteria* and decreased *Firmicutes* and *Bacteroidetes* in preterm infants (31). In the same model, rectal transplantation with fecal microbiota from healthy term piglet donors decreased the NEC incidence in formula-fed preterm piglets, indicating important microbial effects in NEC pathogenesis (32). When it comes to feeding, colonic microbiota did not differ between colostrum- and formula-fed 5-day-old pigs (12), while the diversity was increased and *Campylobacter* was decreased in colostrum-fed 11-day-old pigs (18). Whether the beneficial effects of colostrum attribute (or partly) to gut microbiota remains to be investigated.

Despite the minimal effects on macroscopic NEC lesions in the SI region, structural, and functional parameters were largely impaired by formula feeding both in short- and longer-term studies. Similar to our previous results, formula feeding, irrespective if given in large volumes in a rapid advancing manner or small volumes in a slow advancing manner, induces villus atrophy (decreased villus height and increased crypt depth), digestive dysfunction, and increased permeability (12, 16, 22). In a previous study, we showed that formula-induced villus atrophy in preterm pigs may be due to increased enterocyte apoptosis (33), which is supported by the upregulation of an apoptosis gene, *CASP3* in the formula-fed pigs in this study. Bioactive factors found in high amounts in colostrum, such as lactoferrin, TGF-β2, and IGF-I, have been shown to attenuate epithelial apoptosis in cell and animal models (34–36). In this study, colostrum feeding was able to prevent and restore formula-induced impairment in terms of villus atrophy, digestive and absorptive functions, and permeability, which may relate to its apoptosis-suppressed effects supported by the similarly downregulation of CASP3 in the CF and FC groups, relative to the FF group.

Furthermore, the impaired gut structure and increased permeability may allow bacteria and/or toxins to adhere to mucosae and translocate, leading to local inflammation or even systemic immune stimulation. Fluorescence *in situ* hybridization staining showed that colostrum-fed pigs had less bacterial adhesion to SI mucosa relative to the formula-fed ones (18, 19). The close contact with bacteria and its toxins induces mucosal inflammation and further deteriorate gut integrity. In the current study, gut innate immunity- and hypoxia-related genes such as *LBP, MYD88, IL8*, and *C3* were upregulated in formula-fed pigs as shown in previous studies (12, 17, 18, 37). *LBP* regulates the transcription of LPS binding proteins in enterocytes, which binds and deactivates LPS during the acute phase response to endotoxin (38). The upregulation of MYD88 (an adaptor protein activates the transcription of NF-κB), IL8 (a key neutrophil chemoattractant and activator), and C3 (a key component in complement system facilitating phagocytosis or direct lysis of targeted cells) designated the activation of Toll-like receptor pathways and enhanced neutrophil recruitment and phagocytosis in response to the inflammatory challenge (18, 39). These genes were less affected by diets in 9-day-old pigs, which may reflect a reduction in inflammatory challenge to the mucosa over time.

Mucosal inflammation limits the oxygen availability and may lead to secondary hypoxia in the epithelial cells and subsequent inflammatory lesions (40). Upregulated HIF1A (the master transcriptional regulator of hypoxia stress) has been observed in human NEC tissues (41), and its downregulation by colostrum feeding might be an important strategy in preventing inflammatory lesions in the gut. Our results showed that the initial downregulation of HIF1A by colostrum feeding persisted on day 9 even when switched to formula feeding for the last 4 days. These gut effects of colostrum may attribute to the high levels of bioactive compounds that neutralize bacteria and toxins (e.g., lysozyme, lactoferrin, Igs), stimulate intestinal function and integrity (e.g., IGF-I, TGF-β, lactoferrin), or attenuate inflammation, apoptosis, and oxidative and hypoxic stress (e.g., lactoferrin, TGF-β) (33, 42, 43).

Relative to exclusive formula feeding, initial feeding of colostrum for 4 days influenced the development of T-cell subpopulation on day 9, even when switched to formula feeding, including lowered T_h_ and increased T_reg_ and DN frequencies. Lowered T_h_ frequency has also been observed in breast-fed infants compared with formula-fed ones, suggesting less systemic immune activation in MM-fed infants (44). The corresponding increase in T_reg_ reflected the development of immune tolerance toward excessive inflammatory response in the circulation. This effect of colostrum on systemic immune regulation may function via the gut by dampening mucosal proinflammatory response immediately after birth and potentially reducing gut translocation of bacteria and toxins. It is noteworthy that the flow cytometer used in this study can maximally detect four colors, which limited our choice to staining for CD3, CD4, CD8, and Foxp3. Although CD4^+^CD25^+^Foxp3^+^ are often used as T_reg_ markers, it has been reported that T_reg_ can be present in both CD25^+^ and CD25^−^ forms with similar capacity of producing anti-inflammatory cytokines (IL-10 and TGF-β) in pigs (45). Therefore, it is considered sufficient, at least in pigs, to use CD3^+^CD4^+^Foxp3^+^ as markers for T_reg_. Similarly, NK cells in pigs are CD3^−^ (46, 47) and therefore are not categorized as T_c_ cells (stained as CD3^+^CD4^−^CD8^+^) in this study. However, some subsets of NKT cells in pigs may express CD8, so the T_c_ cells in this study may contain some NKT cells, although with very low frequency [0.01–1% of total CD3^+^ T cells (48)].

In the current study, we aimed to investigate the effects of intact colostrum as a food matrix, rather than investigating individual bioactive components separately. In a recent large randomized controlled trial with 2,203 preterm infants, oral supplementation of bovine lactoferrin (the most promising single bioactive milk compound) failed to reduce the risk of late-onset infection and other morbidities (49). Possibly, these bioactive factors found in milk may function in synergy, and an isolated factor may not be effective. Nevertheless, unmodified colostrum should not be used as the sole food for infants, as it contains unbalanced nutrient composition relative to human milk. For example, colostrum contains high proteins, low carbohydrates, and different levels of vitamins and minerals compared with MM and formula (14). On the other hand, severely modified colostrum will lose its bioactivity and may exert detrimental effect in preterm infants (50). In conclusion, our study underlines the importance of initial enteral feeding and how initial feeding shapes the developmental trajectory of intestinal health and systemic immunity in preterm neonates. Although colostrum also had some therapeutic effects to repair the detrimental effects induced by formula feeding, it was more restricted in the digestive system with less strength. Thus, bovine colostrum is suggested to be used as the initial feeding to prepare the digestive and immune systems in very preterm infants for the later consumption of formula in resource-limited hospitals, when MM is not available immediately after birth.

## Data Availability Statement

The datasets generated for this study are available on request to the corresponding author.

## Ethics Statement

The animal study was reviewed and approved by Danish National Committee on Animal Experimentation.

## Author Contributions

YL, XP, DN, and PS designed research and interpreted the data. YL and XP conducted the animal experiments. XP, SR, and AM conducted lab analyses. YL analyzed data and wrote the paper. All authors critically revised and approved the final manuscript.

### Conflict of Interest

The University of Copenhagen holds a patent on the use of colostrum for pediatric patients. PS is listed as a sole inventor but has declined any share of potential revenue arising from commercial exploitation of such a patent. The remaining authors declare that the research was conducted in the absence of any commercial or financial relationships that could be construed as a potential conflict of interest.
